# Phylogeographic analyses of a widely distributed *Populus davidiana*: Further evidence for the existence of glacial refugia of cool‐temperate deciduous trees in northern East Asia

**DOI:** 10.1002/ece3.4755

**Published:** 2018-12-11

**Authors:** Zhe Hou, Zhaoshan Wang, Zhanyang Ye, Shuhui Du, Shuyu Liu, Jianguo Zhang

**Affiliations:** ^1^ State Key Laboratory of Tree Genetics and Breeding, Key Laboratory of Silviculture of the State Forestry Administration, Research Institute of Forestry Chinese Academy of Forestry Beijing China; ^2^ Collaborative Innovation Center of Sustainable, Forestry in Southern China Nanjing Forestry University Nanjing China; ^3^ College of Forestry Shanxi Agriculture University Taigu Shanxi China

**Keywords:** East Asia, molecular variation, northeast refugia, Quaternary glaciations, temperate forest

## Abstract

Despite several phylogeographic studies had provided evidence to support the existence of glacial refugia of cool‐temperate deciduous trees in northeast China, the species used in these studies were limited by the species ranges, which could not exclude the possibility that northern populations were the colonists from southern refugial populations during the last glacial maximum (LGM). Here, we estimated the nucleotide variation in *Populus davidiana*, a widespread species distributed in Eurasia. Three groups in northeast, central, and southwest China were constructed according to the simulation results from SAMOVA, composition of chloroplast haplotypes and structure results. We revealed that the northeast China had endemic haplotypes, the haplotypes and nucleotide diversity in northern regions were not lower than that in southern China, and this species has not experienced population expansion base on the estimation of Bayesian skyline plots. Ecological niche modeling (ENM) indicated that the northeast China had a high suitability score during the last glacial maximum. The combined evidence clearly demonstrated that northeastern and southwestern refugia were maintained across the current distributional range of *P. davidiana* during the LGM. The genetic differentiation between these two refugia might be mainly caused by differences of climate among these areas. The phylogeographic analyses of a widely distributed *P. davidiana* provided robust evidence to clarify the issue of refugia in northeast China, and these results are of great importance for understanding the influence of Quaternary glaciations on the distribution and evolution of species in East Asia.

**Abbreviations**
DB (Dongbei): the northeast of China
HB (Huabei): the central China
NF (Nanfang): the southwest of China

## 1 INTRODUCTION

Climatic oscillations have profoundly affected the current distributions and genetic structures of many plant and animal species in the Northern Hemisphere in the past three million years (Hewitt, 2004; Qiu, Fu, & Comes, 2011). In Europe and North America, it is now well appreciated that the advance and retreat of ice sheets through multiple glacial cycles, especially the last glacial maximum (LGM, 21,000–18,000 years ago), strongly impacted the distribution and genetic diversity of many temperate species (Hewitt, 2004). In contrast, mainland China had never been directly affected by extensive and unified ice sheets although it experienced several climatic oscillations throughout the Quaternary (Harrison, Yu, Takahara, & Prentice, 2001; Qiu et al., 2011; Sun, Mu‐Lin, & Pan, 1977). Moreover, mainland China stretches across tropical, subtropical, temperate, and cold temperate zones from south to north and contains a rich variety of terrains, being the region that harbors the most diverse temperate flora in the world and has a higher level of biodiversity than Europe and North America (Qian & Ricklefs, 1999). Some ice‐age refugia have been confirmed in the southern regions of China (Chen, 2011), where the tropical areas and the presence of great mountainous regions contributed to the maintenance and possibly continuous diversification of the more tropical elements of the Chinese flora (Qian & Ricklefs, 1999). During the Quaternary glaciations, although most of regions in China were never covered by ice sheets, dramatic climatic oscillations led to the extinction of many plants in the north of China (Cheng, Hwang, & Lin, 2005; Lu, Peng, Cheng, Hong, & Chiang, 2001; Shen et al., 2005; Zhang, Chiang, George, & JQ & ABBOTT, R., 2005), and whether these areas have glacial refugium of cool‐temperate deciduous trees is a continuing debate. This is of great importance for understanding the influence of Quaternary climate fluctuations on the distribution and evolution of species in China and in Asia.

Some authors have pointed out that during the LGM, temperate forests, steppe, and even desert vegetation which covered the areas currently dominated by coniferous and deciduous forests would have retreated southward below 30°N in the northwest and reaching 25°N in eastern China, and then at the warm and wet interglacials, they recolonized the previously uninhabitable northern regions (Cao, Herzschuh, Ni, Zhao, & Boehmer, 2015; Ni, Harrison, Prentice, Kutzbach, & Sitch, 2006; Yu et al., 2000), such as the thermophilous (*Castanea*, *Castanopsis*, *Cyclobalanopsis*, *Fagus*, *Pterocarya*) and eurythermal (*Juglans*, *Quercus*, *Tilia*, *Ulmus*) broad‐leaved tree taxa in tropical or subtropical areas of China (Cao et al., 2015; Ni et al., 2006; Yu et al., 2000). Other researchers, however, rejected this hypothesis and suggested that the cold climate and ice sheets might have reduced the distribution of most forest species, providing evidence that multiple LGM refugia may have allowed species to persist across northern China. Bai, Liao, and Zhang (2010) examined the phylogeography of *Juglans mandshurica*, a temperate deciduous walnut tree distributed in northern and northeastern China, and found two independent refugia in northern China during the LGM. This was contrary to the inference of the southward retreat and northward expand of temperate forests. Phylogeographic studies of organisms from North China and Northeast China suggested that, during the LGM, cool‐temperate deciduous tree species in East Asia persisted within their modern northern range (Tian et al., 2009; Zeng, Wang, Liao, Wang, & Zhang, 2015).

Despite several phylogeographic studies that provided evidence supporting the existence of glacial refugia in northeast China, some questions are still poorly solved. Hewitt pointed out that while species were expanding northwards, southern populations would die out as the southern edge of the species’ tolerance range also moved north during the postglacial period (Hewitt, 1999). This phenomenon could also lead to the distribution patterns of those species in north and northeast China noted above. Nevertheless, the previous studies could not rule out this possibility because all the species they used were restricted to north and northeast China, the Korean Peninsula, Japan, and the Russian Far East. So, a species widely distributed across mainland China is needed to solve this problem through the phylogeographic study.

*Populus davidiana* Dode (Salicaceae), a temperate deciduous tree distributed continuously in mainland China, Mongolia, Korea, and the Far East of Russia, which is a keystone species in boreal forest communities. *P. davidiana* is highly resistant to cold, drought, and barren soils. Natural populations mainly grow on slopes, ridges, and gullies, often forming a small area of pure or mixed forest with other tree species, so the influence of human activities on their distribution is relatively small. Therefore, *P. davidiana* is an excellent model to shed light on the phylogeographical history of Asian trees.

To verify the two possible origins of cool‐temperate deciduous trees in northeastern China, it is necessary to obtain a widely distributed species’ population structure and genetic diversity distribution. In this research, we used both nuclear and cpDNA sequences and ecological niche models (ENMs) to identify the phylogeographic patterns of *P. davidiana*. We specifically aimed to address: (a) whether or not glacial refugia existed across northeast China, and (b) reconstruct the Quaternary history of the species through evaluating the distribution patterns of chloroplast haplotypes and nuclear gene polymorphisms within and among populations of *P. davidiana*.

## 2 MATERIAL AND METHODS

### 2.1 Population sampling

**Figure 1:**
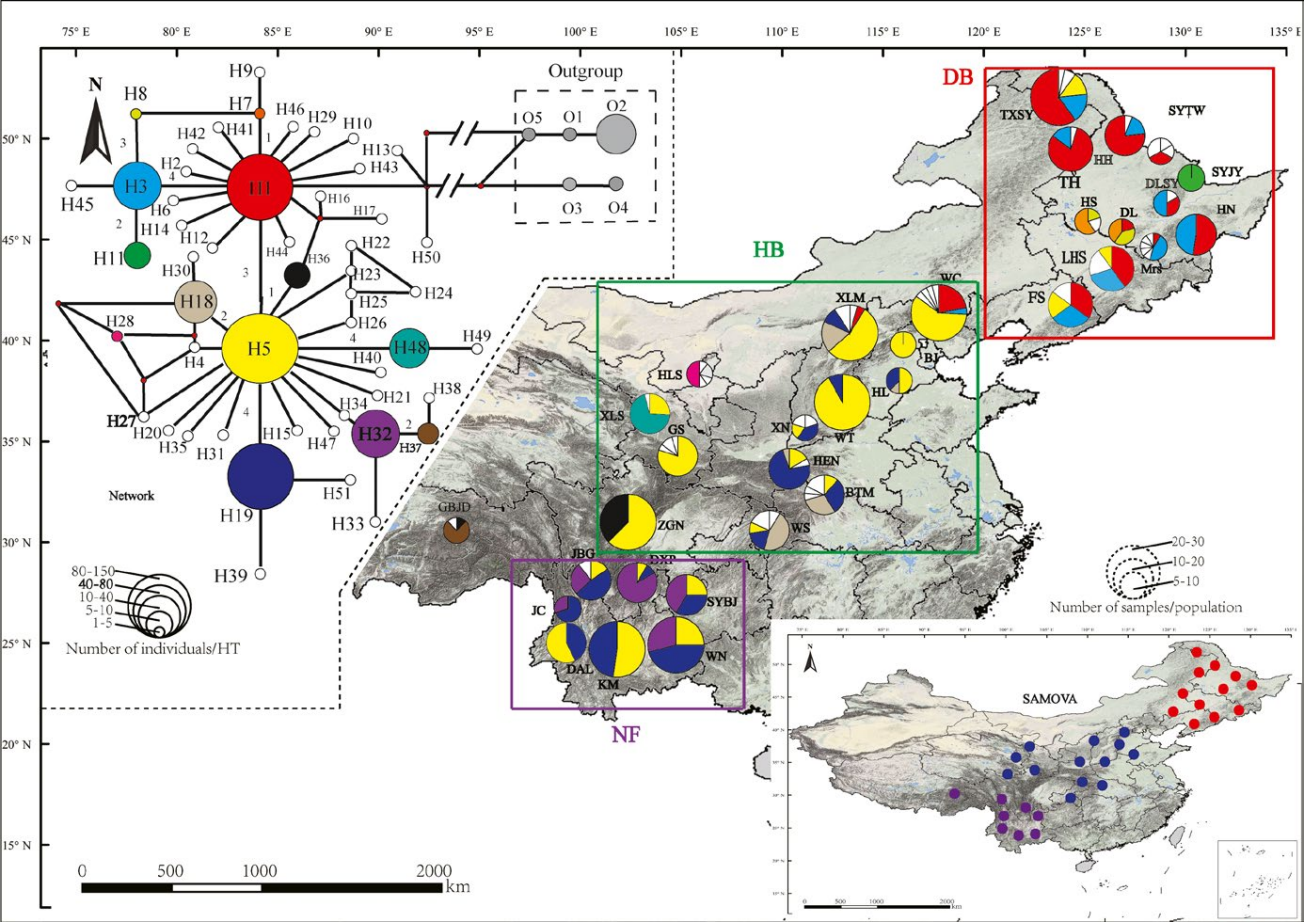
Geographic distribution of the 32 sampled populations of *P. davidiana*. Distribution frequencies of cpDNA haplotypes, with networks of the cpDNA haplotypes constructed using NETWORK 5.0.0.0. Colored haplotypes are shared by two or more populations, and blank ones are private haplotypes. Haplotypes O1–O5 appear exclusively in outgroups. The sizes of circles in the network are proportional to the observed number of individuals in the haplotypes, and the sizes of the circles on the map are proportional to the population sizes of sampling locations, the number on the line show the number of mutations between haplotypes in the network, bottom right dots of different colors represent the three population groups identified by SAMOVA

A total of 502 individuals of *P. davidiana* were sampled from 32 natural populations throughout its distribution range in mainland China (Figure 1). These populations covered most of the species range. The population code, location, and sampling size are shown in Supporting information Table S1. The distance between any two sampled trees in the same population was at least 100 m to prevent repeated sampling because of the root sprout reproduction of *P. davidiana*. Leaf tissues were silica‐dried and stored at room temperature until DNA extraction.

### 2.2 DNA extraction, amplification, and sequencing

Total genomic DNA was isolated from ~25 mg silica‐gel‐dried leaf from each individual of 32 natural populations using a modified CTAB method (Doyle, 1987). Based on the study of Du et al. (2015), three cpDNA fragments (*trnK*, *trnL‐trnF*, and *atpI*) and six single‐copy nuclear loci (DSH3, DSH5, DSH6, DSH7, DSH12, and DSH21) were used to amplify and sequence 8 to 30 *P. davidiana* trees of the 32 populations. Primers used are listed in Supporting information Table S2. The polymerase chain reaction (PCR) and sequencing were based on the research of Du et al. (2015). We sequenced (ABI 3730 DNA ANALYZER) the PCR amplicons from both ends. For samples with sequence peaks or sequence failure, we sequenced after cloning. We used the software DNAsp 5.10 (Librado & Rozas, 2009) to phase the sequences with heterozygote sites. Other sequences including *P. davidiana* and *Populus tremuloides *(Salicaceae) were downloaded from GenBank (accession numbers KP887111–KP887162). The new sequences were deposited in GenBank under accession numbers (MG287147–MG288502, MG345155–MG346171, MG426214–MG427048).

### 2.3 Data analysis

#### 2.3.1 Distribution and variation of cpDNA haplotypes

The assembled contigs of each individual were aligned using CLUSTALX (Thompson, Gibson, Plewniak, Jeanmougin, & Higgins, 1997) and refined manually in BioEdit (Hall, 1999). DnaSP 5.10 (Librado & Rozas, 2009) was employed to extract the cpDNA and nuclear haplotypes (Librado & Rozas, 2009). In order to reliably infer population history and relationships among individuals, a haplotype network was constructed using Network version 5.0.0.0 (Bandelt, Forster, & R HL, A., 1999) (available at http://www.fluxus-engineering.com) following the median‐joining method, in which all indels were not considered.

A spatial analysis of molecular variance (SAMOVA) was performed for cpDNA sequence matrices to define the number of population groups using SAMOVA 2.0 (Dupanloup, Schneider, & Excoffier, 2010). Based on a simulated annealing procedure, the SAMOVA algorithm iteratively seeks the population composition of a user‐defined number of groups (K) that aims to maximize the proportion of total genetic variance (FCT) due to differences between population groups (Dupanloup et al., 2010). The software was run with default parameters. The number of initial conditions was set to 500 with *K* = 2–10, and FCT was used to determine the most likely *K*.

Two measures of population differentiation, *G*_ST_ and *N*_ST_, were calculated using the program PERMUT (Pons & Petit, 1996). We inferred phylogeographic structure by testing whether *N*_ST_ was significantly larger than *G*_ST_ using a permutation test with 1,000 random permutations of haplotypes across populations. A significantly higher *N*_ST_ over *G*_ST_ usually indicates the presence of phylogeographic structure and that the populations are strongly differentiated genetically. If *N*_ST_ is equal to *G*_ST_, then it is likely the haplotypes are phylogenetically equivalent. Finally, if *N*_ST_ is significantly smaller than *G*_ST_, then the relative geographic distribution of haplotypes is likely unrelated to their genetic distances.

#### 2.3.2 Molecular diversity of each locus in each region of *P. davidiana*

The molecular diversity indices, including a number of segregating sites (*S*) and haplotypes (*H*), haplotype diversity (*H*_d_), nucleotide diversity parameters (π), Watterson's *θ*_w_ (Watterson, 1975), and the minimum number of recombination events (*R*_m_), were estimated for the regions (the composition of these regions are listed in the Table 2) at each locus using DnaSP (Librado & Rozas, 2009). Tajima's D (Tajima, 1989) and Fu and Li's D* and F* (Fu & Li, 1993) were applied to determine whether a locus is evolving neutrally and is therefore appropriate for phylogeographic study (Caicedo & Schaal, 2004). The geographical distributions of haplotypes were labeled on a relief map of China using DIVA‐GIS (Hijmans et al., 2004). Genetic differentiation among regions (*F*_ST_) for each nuclear locus was also calculated in Arlequin 3.5.2.2 (Excoffier, Laval, & Schneider, 2005).

#### 2.3.3 Genetic structure

To identify the possible genetic structure of *P. davidiana* populations, the program STRUCTURE version 2.3.4 (Falush, Stephens, & Pritchard, 2003) was run, assuming a preassigned number of genetic clusters (*K*) ranging from 1 to 10 and 20 replicate runs were carried out for each K. We used the admixture model and the correlated allele frequencies model to estimate the genetic structure of *P. davidiana*. All runs included a burn‐in period of 100,000 iterations, following 1,000,000 Markov Chain Monte Carlo repetitions with the aim of ensuring the reproducibility of the STRUCTURE results (Gilbert et al., 2012). The final posterior possibility of *K*, Delta *K* (Δ*K*), Ln *P* (*K*), and the rate of change of Ln *P* (*K*) between successive *K* values (Evanno, Regnaut, & Goudet, 2005) were estimated using Structure Harvester (Earl & Vonholdt, 2012) to determine the most possible number of clusters.

To estimate the relative contribution to molecular variance within and among the defined three groups (the DB, HB and NF, excluding the GBJD population), which were obtained from the STRUCTURE results, an analysis of molecular variance (AMOVA) was performed in Arlequin 3.5.2.2 (Excoffier, Laval, & Schneider, 2007) based on the cpDNA and five nuclear single‐copy genes.

#### 2.3.4 Coalescent inferences of demographic history

To reconstruct the demographic changes of three identified groups over time, the historical demographic dynamics of *P. davidiana* were inferred from Bayesian skyline analyses implemented in BEAST 1.2 (Drummond, Rambaut, Shapiro, & Pybus, 2005). This recently developed coalescence‐based approach utilizes standard MCMC sampling procedures to evaluate the posterior probability distribution of effective population size during the intervals under a GTR substitution model (Pybus, 2006). We used both nuclear loci and cpDNA loci sequences for this analysis. Independent MCMC analyses were run for 1 × 10^8^ steps, sampling every 105 steps and discarding 1,000 samples as burn‐in. For each clade, multiple analyses were performed with different random seeds to test for convergence, and results from replicate runs were pooled using LogCombiner and visualized skyline plots with TRACER 1.4 (Drummond et al., 2005).

We used Bayesian analysis to estimate the divergence times between pairwise groups (Drummond et al., 2005). Simultaneously to estimate theta (*θ*, measured as 2*N*_e_ *μ*, where *N*_e_ is the effective population size and *μ* is the mutation rate per sequence per generation), population divergence time (*t*_pop_, measured as *t*/*N*_e_, where *t* is the time since population divergence). Markov chain simulation for 5 × 10^7^ steps, where the first 5 × 10^6^ were discarded as burn‐in; and uniform prior distributions from 0 to 5 for *t*_pop_. Convergence was determined by assessing the consistency of model values for each of the parameters across three runs. The modes of the posterior distribution for both *T* and *θ* were used to estimate divergence times between pairwise clades. We assumed a mutation rate of 2.5 × 10^−9^ per site per year and a generation time of 15 years in *Populus* (Koch, Haubold, & Mitchell‐Olds, 2000).

#### 2.3.5 Pleistocene and present ecological predictive models

The global climate database from WorldClim (Hijmans, Cameron, Parra, Jones, & Jarvis, 2005) was used to infer the potential distribution of *P. davidiana* in the present, the last inter glacial (LIG), and the last glacial maximum (LGM) using MAXENT v.3.3.335 (Phillips & Dudík, 2008). For this species, the distribution model was generated using 19 bioclimatic parameters (Table S3) for the current, LGM, and LIG climate of the collection localities. To reduce over‐fitting of ecological niche modeling, we conducted Pearson's correlation for environmental variables using the methods of Sheppard (Zheng, Fan, et al., 2017). A total of 230 distribution records were used for modeling, which included the sampling sites in this study supplemented by the Chinese Virtual Herbarium (http://www.cvh.org.cn/). Climate estimates for the LGM were provided by the Community Climate System Model (CCSM) and the Model for Interdisciplinary Research on Climate (MIROC) 3.2 (http://www.pmip2.cnrs-gif.fr) at 2.5 arc‐min resolution, as well as climate estimates for the current time (http://www.worldclim.com). In addition, we projected the model to the LIG (~120,000 to 140,000 years BP) using the climate model of Otto‐Bliesner *et al.* (Otto‐Bliesner, 2006). Binomial tests of omission were carried out by randomly selecting 25% of the occurrence locations as test data and using 10,000 randomly chosen pixels from the study region as random instances.

## 3 RESULTS

### 3.1 cpDNA haplotype variation and distribution

The total alignment length of the cpDNA sequence was 3,263 bp. Not considering indels, 41 polymorphic sites (14 singleton variable sites and 27 parsimony informative sites) were contained in the cpDNA sequences of the *P. davidiana* samples.

Fifty‐one haplotypes were found in the combined cpDNA sequences of *P. davidiana*. Haplotypes distributed in each population are presented in Figure 1. Among the 51 haplotypes, only 11 were shared among populations, while the others were characteristic of single population. Six haplotypes were common (frequency > 5%): H5 (18.3%), H1 (17.5%), H19 (14.2%), H48 (9.2%), H32 (8.9%), and H3 (5.8%), and the frequencies of the remaining 50 rare haplotypes ranged from 0.3% to 3.3%. Among the 32 populations of *P. davidiana*, two (SYJY and BJ) were fixed by a single haplotype (H5 and H11), while the remaining 31 were polymorphic.

Simulation results from SAMOVA suggested that FCT increased greatly from *K* = 2 to *K* = 5 and then reached a plateau at *K* > 5. However, if single‐population clusters were left out, as is usual in SAMOVA, we ended up with *K* = 3 as the optimal number of population groups (Figure 1).

According to the simulation results from SAMOVA and composition of chloroplast haplotypes in these populations, three regions were structured: we used DB, HB, and NF to represent the northeast of China, the central China, and the southwest of China, respectively (Figure 1). Haplotypes H3 appeared in the DB and HB region and its frequency in DB (20.0%) was higher than in HB (3.2%). This phenomenon was also found for H1 (46.3% in DB and 11.3% in HB). According to the network of chloroplast haplotypes, H5 located at the center of the gene tree and was geographically widespread. Both the NF and HB regions contained Haplotype H19. Haplotype H32 only existed in the NF region and Haplotype H18 only existed in the HB region.

Based on the cpDNA data, the relationship between haplotype spectrum history and the geographical distribution of the populations in northeast, middle, and southwest of China were tested. The results showed that between northeast and middle of China: *N*_ST_ » *G*_ST_ (*N*_ST_ = 0.505, *G*_ST_ = 0.204); and between northeast and southwest: *N*_ST_ » *G*_ST_ (*N*_ST_ = 0.604, *G*_ST_ = 0.212; Table 1).

**Table 1:** The relationship between haplotype spectrum history and the geographical distribution of the three regions

Regions	*N*_ST_	*G*_ST_	*p *value
DB‐HB	0.505	0.204	**
HB‐NF	0.180	0.135	–
DB‐NF	0.604	0.212	**

Note

DB: the northeast of China; HB: the central China; NF: the southwest of China; *p* value: Significant level, **Extremely significant; –, not significant.

### 3.2 Genetic diversity and differentiation

The nucleotide diversity of each locus is shown in Table 2. The average nucleotide diversities of the three groups across loci were π = 0.0031–0.0040, *θ*_w_ = 0.0038–0.0053. As expected, the sequence variation of cpDNA was lower than that of the nuclear loci, reflecting the lower mutation rate observed in the chloroplast genome of plants. The mean haplotype diversity (*H*_d_) ranged from 0.571 to 0.656 for the six nuclear loci. The mean diversity levels of nucleotides and haplotypes of the nuclear loci in the DB and HB were slightly higher than in the NF region (Table 2). For single locus tests, most loci displayed negative, but not significant Tajima's D. However, the results for the DSH21 locus deviated significantly from neutrality both in the species and most of the regions, suggesting that this locus may have been subject to selection and will be excluded from further analysis. The minimum number of recombination events (*R*_m_) of the nuclear loci ranged from 1 to 12 in the species, implying high levels of outcrossing. As expected, the sequence variation of cpDNA was lower than the nuclear loci, which was reflected by the lower mutation rate in the chloroplast genome of plants and the lower effective population size of the cpDNA.

**Table 2:** Nucleotide diversity and neutral tests of each locus in each region of *P.davidiana*

Regions	LOCUS	*N*	*L*	*H*	*S*	π	*θ*_w_	*D* *	*F* *	Tajima's *D*	*R*_m_	*H*_d_
DB	DSH3	166	606	22	17	0.0035	0.0046	0.47	0.05	−0.62	3	0.746
	DSH5		460	24	17	0.0070	0.0061	0.47	0.51	0.36	8	0.869
	DSH6		542	50	32	0.0042	0.0097	−0.29	−1.01	−1.58	6	0.882
	DSH7		530	12	12	0.0033	0.0037	−0.02	−0.13	−0.26	1	0.529
	DSH12		548	17	12	0.0026	0.0036	−0.74	−0.85	−0.65	2	0.712
	DSH21		747	12	17	0.0005	0.0037	−1.87	−2.43*	−2.24**	1	0.201
	**Mean**		572	23	18	0.0035	0.0052	−0.33	−0.29	−0.55	3	0.656
	*trnK*		2,110	15	23	0.0006	0.0024	−5.59**	−5.13**	−2.24**	0	0.622
	*trnL‐trnF*		488	5	4	0.0001	0.0015	−1.94	−2.16	−1.53	0	0.026
	*atpI*		968	9	13	0.0001	0.0026	−3.46**	−3.40**	−1.80*	1	0.488
	**Mean**		1,188	9	13	0.0002	0.0022	−1.94	−2.16	−1.53	0	0.379
HB	DSH3	192	606	36	29	0.0059	0.0074	−1.48	−1.34	−0.62	9	0.884
	DSH5		460	26	18	0.0069	0.0075	−0.74	−0.64	−0.21	6	0.755
	DSH6		542	66	28	0.0054	0.0080	0.60	−0.24	0.85	12	0.882
	DSH7		530	12	11	0.0037	0.0032	0.55	0.55	0.31	2	0.525
	DSH12		548	10	8	0.0015	0.0022	1.16	0.59	−0.69	2	0.378
	DSH21		747	11	18	0.0005	0.0037	−5.51**	−5.04**	−2.14**	0	0.282
	**Mean**		572	26	18	0.0040	0.0053	0.018	−0.216	−0.072	5	0.62
	*trnK*		2,149	19	25	0.0009	0.0022	−4.06**	−3.77**	−1.77*	0	0.848
	*trnL‐trnF*		488	2	1	0.00003	0.0004	−2.19	−2.12	−0.95	0	0.01
	*atpI*		1,006	13	12	0.0006	0.0024	−1.34	−1.84	−1.87*	0	0.360
	**Mean**		1,214	11	12	0.0005	0.0017	−1.77	−1.98	−0.95	0	0.406
NF	DSH3	112	606	7	6	0.0018	0.0017	1.06	0.88	0.14	0	0.488
	DSH5		458	17	16	0.0047	0.0058	0.41	0.07	−0.49	4	0.774
	DSH6		541	46	21	0.0055	0.0065	0.77	0.34	−0.41	5	0.820
	DSH7		529	9	11	0.0038	0.0035	−0.12	0.02	0.24	0	0.632
	DSH12		548	17	12	0.0026	0.0037	−0.70	−0.83	−0.67	1	0.678
	DSH21		747	3	7	0.0001	0.0016	−4.65**	−4.42**	−1.95*	0	0.035
	**Mean**		571	16	12	0.0031	0.0038	0.28	0.096	−0.24	1	0.571
	*trnK*		2,173	5	5	0.0007	0.0005	1.03	1.25	1.13	0	0.689
	*trnL‐trnF*		421	3	2	0.0011	0.0009	0.68	0.67	0.33	0	0.437
	*atpI*		950	1	0	0.0000	0.0000	–	–	–	–	0.000
	**Mean**		1,181	3	2	0.0006	0.0005	–	–	–	–	0.375

Notes

D*: Fu and Li's D* test statistic; DB: the northeast of China; F*: Fu and Li's F* test statistic; H: number of haplotypes; HB: the central China; *H*_d_, haplotype diversity; L: length of each locus; *N*: number of samples from each region; NF: the southwest of China; S: number of segregating sites; π: nucleotide diversity; *R*_m_: minimum number of recombination events; *θ*_w_: nucleotide diversity; ^*^ *p* < 0.05; ^**^ *p* < 0.02;^***^ *p* < 0.01; the sequencing quality of DLSY is bad for the 6 nuclear gene, so we do not consider the population in the next analysis.

The average haplotype diversity ranged from 0.375 to 0.406 and nucleotide diversity varied between 0.0002 and 0.0005. The mean diversity levels in the DB and HB were slightly higher than those in the NF region (Table 2).

The *F*_ST_ analysis indicated 93.3% of the pairwise *F*_ST_ values were significantly different (*p* < 0.01) and ranged from 0.0069 to 0.5820 for the five nuclear loci (Table 3).

**Table 3:** Pairwise comparisons of *F*_ST_ between 3 regions of *P. davidiana*

Loci	Regions	DB	HB	NF
DSH3	DB	0		
	HB	0.0359**	0	
	NF	0.2631**	0.1200**	0
DSH6	DB	0		
	HB	0.0577**	0	
	NF	0.5820*	0.3841*	0
DSH12	DB	0		
	HB	0.3879**	0	
DSH5	NF	0.2811**	0.0069	0
	DB	0		
	HB	0.2371**	0	
DSH7	NF	0.0954**	0.5508**	0
	DB	0		
	HB	0.3492**	0	
	NF	0.3400**	0.1404**	0

Notes

DB: the northeast of China; HB: the central China; NF: the southwest of China; ^*^ *p* < 0.05; ^**^ *p* < 0.01.

### 3.3 Genetic structure

**Figure 2:**
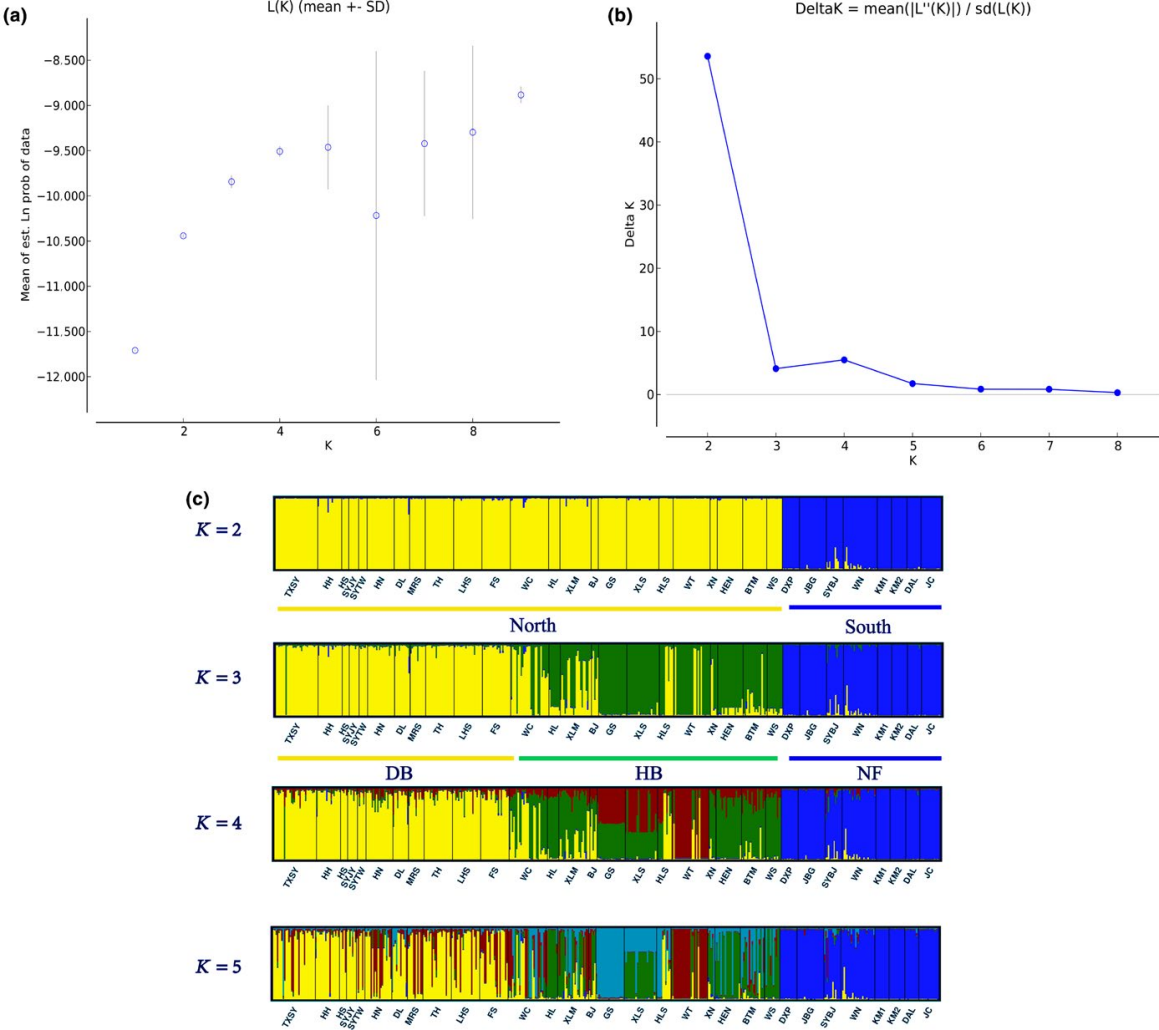
The genetic structure of *P. davidiana* constructed using STRUCTURE. DB: the northeast of China; HB: the central China; NF: the southwest of China; *K* was estimated using (a) the posterior probability of the data given each *K* (20 replicates), (b) the distribution of Δ*K*, and (c) the clusters were detected from STRUCTURE analysis

The structure result indicated that the mean estimated logarithm of probability of the data, L (*K*), increased linearly from *K* = 1 to *K* = 2, and then increased slowly up to *K* = 8, while the highest Δ*K* occurred at *K* = 2 (Figure 2a,b). At *K* = 2, the 32 *P. davidiana* populations were divided into the following two geographical groups: a NORTH group that consisted of all populations in Heilongjiang, Jilin, Liaoning, Hebei, Beijing, Ningxia, Gansu, and Henan Provinces and Wushan county; a SOUTH group consisted of the remaining populations.

When *K* = 3, there was evidence for further population sub‐structuring in the NORTH region, where individuals from populations of DB and HB clustered into two subgroups. With *K* = 4, most individuals of HB region were inferred to be a mixture of two genetic components, showing slight clinal variation with latitude and hybridization. No further structure was found when *K* = 5 (Figure 2c).

AMOVA indicated that variations among groups were significant (*p* < 0.001) and ranged from 14.75% at DSH 7% to 24.51% at DSH12 (Table 4). Genetic differentiations among populations and within populations were significant at all loci (*p* < 0.001). Approximately 12.63% of the total variation was attributed to the variation among populations within group (7.99%–12.67%). Within each group, the average variation components among populations (4.35%–20.87%) were much lower than that within populations (79.13%–95.65%). The pairwise *F*_ST_ values showed strong genetic differentiation among groups (0.251–0.362), and all values were significant (Table 3).

**Table 4:** AMOVA for 3 groups of *P. davidiana*

Locus	Nuclear						Chloroplast			
	DSH3	DSH5	DSH6	DSH7	DSH12	Average	Trnl‐trnf	trnk	atpI	Average
All individuals										
Among groups	23.50	14.78	17.17	14.75	24.51	16.04	55.49	45.50	48.09	49.69
Among populations	12.67	12.42	10.37	12.47	7.99	12.63	9.09	9.45	11.70	10.08
Within groups										
Within populations	63.83	72.80	72.46	72.78	67.50	71.33	35.42	45.05	40.21	40.23
DB										
Among populations	6.21	6.28	4.80	2.11	0.48	4.35	23.51	17.61	15.11	18.74
Within populations	93.79	93.72	95.20	97.89	99.52	95.65	76.49	82.39	84.89	81.25
HB										
Among populations	17.70	20.77	15.90	16.93	25.33	20.87	15.98	11.98	27.54	18.50
Within populations	82.30	79.23	84.10	83.07	74.67	79.13	84.02	88.02	72.46	81.5
NF										
Among populations	40.89	15.59	18.15	30.04	9.95	19.60	0.44	24.40	20.62	15.15
Within populations	59.11	84.41	81.85	69.59	90.05	80.34	99.56	75.60	79.38	84.84
*F*_ST_										
Among groups	0.362	0.272	0.275	0.251	0.325	0.280	0.646	0.563	0.598	0.602

Note

DB: the northeast of China; HB: the central China; NF: the southwest of China; Note: **p < 0.001.

### 3.4 Test of expansion and demographic history

**Figure 3:**
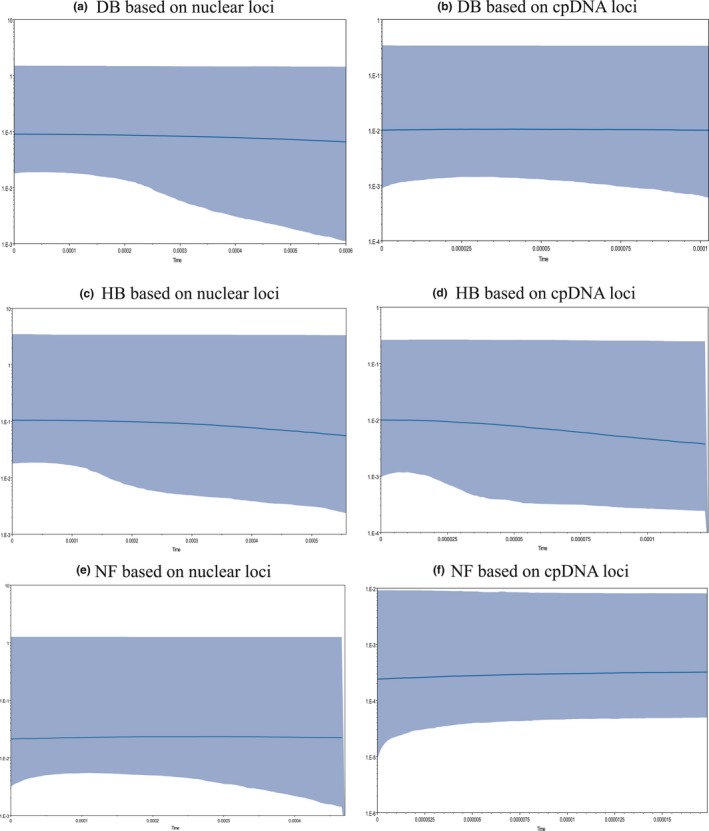
Bayesian skyline plots for the three groups, showing effective population size as a function of time. The upper and lower limits of light blue trend represent the 95% confidence intervals of HPD analysis

Bayesian skyline plots (BSP) revealed effective population size of the three groups (Figure 3). It showed that both DB and NF were nearly at a stable population size. Neither the DB group nor the NF group underwent population expansion. HB group showed a slight trend of increasing population size over time. The estimated divergence time between DB and HB was ~0.09 million years ago, HB and NF was ~0.15 million years ago, DB and NF was ~0.29 million years ago (Table 5).

**Table 5:** Estimates of divergence times (in generations) between pairwise groups

Groups‐pair		*t*_pop_	*θ*	Divergence time (year)
DB	HB	0.86	5.256	88,933
HB	NF	0.95	4.369	152,269
DB	NF	0.53	6.368	289,562

Note

*t*_pop_ are measured in units of 2*N*_e_ *μ*; *θ* = 2*N*_e_ *μ*, and *μ* is the mutation rate per sequence per generation.

### 3.5 Present and past distribution of *P. davidiana*

**Figure 4:**
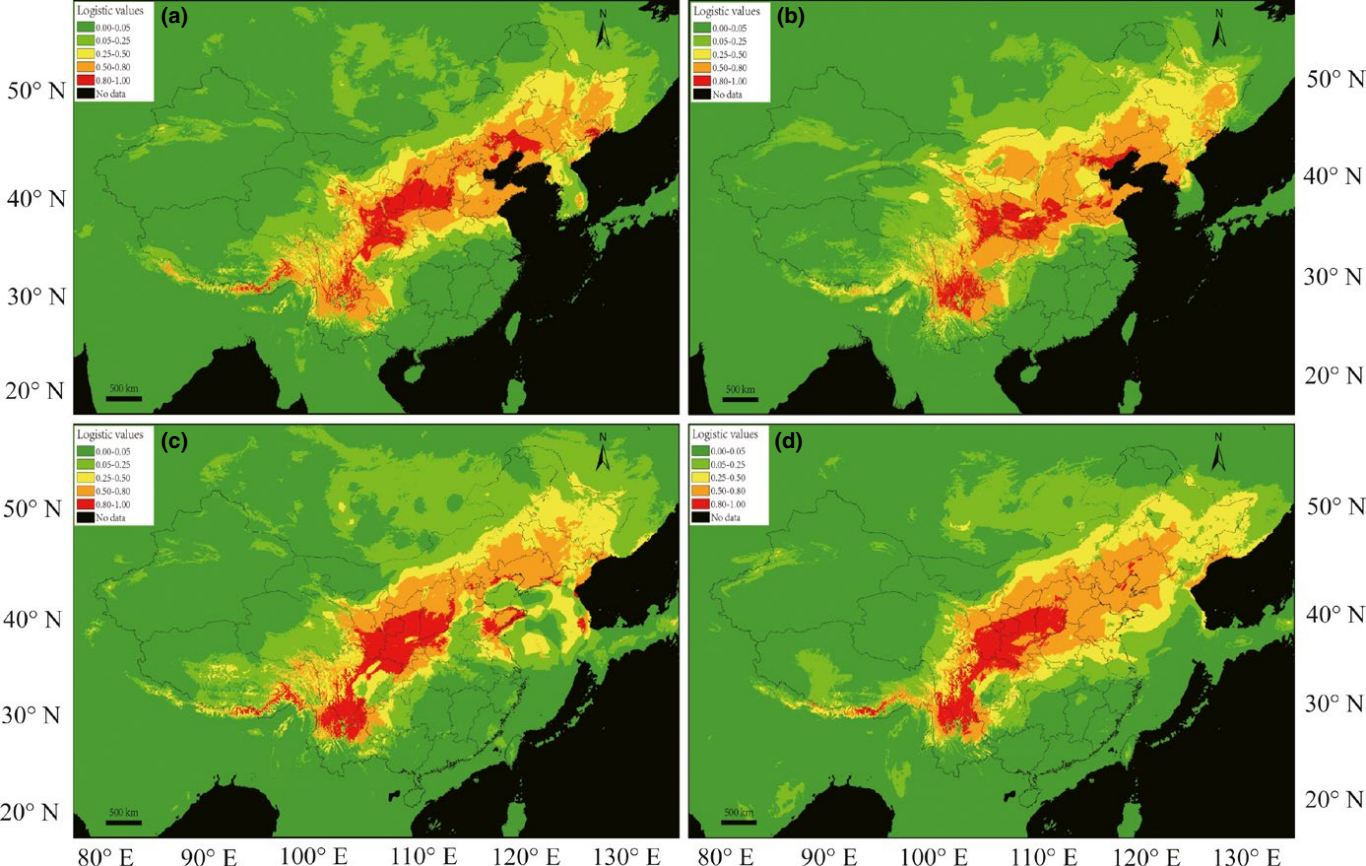
Ecological niche modeling for *P. davidiana* in China. The maps were created using MAXENT and ArcGIS. The strength of prediction is indicated according by the key. Red and orange areas show strong predictions. (a) Regions in China considered suitable for *P. davidiana* under current climate conditions. (b) The paleodistribution model for *P. davidiana* in the LIG (130,000 years ago). (c) The paleodistribution model for *P. davidiana* in the LGM (22,000 years ago) according to CCSM. (d) The paleodistribution model for *P. davidiana* in the LGM (22,000 years ago) provided by MIROC

The ecological niche model of *P. davidiana* had a high predictive power, with an area under the receiving operator curve of 0.987 (*p* < 0.001). The distributional prediction under current climate conditions was a good representation of the extant species distribution between 25°N and 55°N (Figure 4a). The prediction for the LIG was quite similar to the current distributions (Figure 4b). Based on both the CCSM (Figure 4c) and MIROC models (Figure 4d), it was predicted that the suitable habitats were a little smaller than the current distributions during the LGM, especially north of 35°N. But there were still habitats with high suitability scores (>0.80, range from 35°N to 43°N and from 114°E to 122°E) and moderately suitable areas (>0.50, range from 43°N to 46°N and from to 122°E to 130°E) under the CCSM and MIROC models. On the other hand, the predicted distributions in the areas between 25°N and 35°N were similar under the climate of all three periods and revealed two highly suitable distribution areas: the Hengduan Mountains, and areas around the Qinling Mountains.

## 4 DISCUSSION

A variety of forest types are distributed in northeast China, comprising coniferous, broad‐leaved, subalpine, and alpine tundra forest zones at different altitudes (Zheng, Xiao, Guo, & Howard, 2017). Glaciers only occurred at elevations above 2,000 m in this region and adjunct areas in eastern Asia during the glacial cycle in the Pleistocene, while the comprehensive and potentially much larger unglaciated areas may have allowed for the maintenance of smaller refugia (Wei, Niu, Ling, & Cui, 2008).

### 4.1 Glacial refugia in northeastern China

Recently, several phylogeographic studies had proved that there were many glacial refugia in northeast China (Bai et al., 2010; Li‐Jiang et al., 2008; Liu & Harada, 2014). However, northern populations were likely to migrate from the south due to environmental changes during the postglacial period. To verify the two possible origins of cool‐temperate deciduous trees in northeastern China, it is necessary to obtain a widely distributed species’ population structure and genetic diversity distribution. *P. davidiana*, a widely distributed in China from south to north, can be used to verify the two possibilities.

According to the results of cpDNA haplotype variation and distribution, haplotypes H1 and H3 only existed in the DB and HB region and mainly appeared in the DB region (Figure 1) and they were all high‐frequency haplotypes (frequency > 5%). In the haplotype network, the most ancient haplotypes should be located at the center of the gene tree and be geographically widespread, while the most recent haplotypes should be at the tips of the gene tree and be geographically localized (Schaal, Hayworth, Olsen, Rauscher, & Smith, 2010). Based on the network of cpDNA haplotypes, it was clear that H1 and H3 were located at interior nodes of the gene tree and indicate that these two haplotypes were ancient (Schaal et al., 2010), which may indicated that there was a glacial refugium in northeast China. A recent study based on several nuclear and chloroplast loci suggested that the ancestor of *P. davidiana* originated in Northern America and diffused into Eurasia through the Bering land bridge (Du et al., 2015). Geographically, after *P. davidiana* spreading to Asia, it may migrate from northeast China to the south. In particular, the connection between the outgroup and northern haplotypes, while southern haplotypes tend to be derived, is highly suggestive of this. Therefore, it is reasonable that the ancient haplotypes existed in northeast China. This result also supported that the northeast populations were the descendant of the local ancestral refugial populations rather than the colonist from the south populations.

In addition, the number of cpDNA haplotypes in the DB region (16) was not lower than other regions, and this was supported by the mean higher level of haplotype and nucleotide diversity of the cpDNA locus (π = 0.0004, *θ*_w_ = 0.00200–0.00472, *H*_d_ = 0.352, Table 2). If the species re‐colonized the northern regions from southern areas in the process of postglacial expansion, the nuclear gene polymorphism of the northern populations would be lower as alleles and heterozygosity are lost in the process of migration (Hewitt, 2000). In Europe and North America, many studies have reported lower genetic diversity in northern populations that expanded from southern refugial populations (Hewitt, 2000; Soltis, Gitzendanner, Strenge, & Soltis, 1997; Zink, 1996). However, according to the results of genetic diversity, the DB region had a higher mean level of haplotype and nucleotide diversity of nuclear loci (π = 0.0034, *θ*_w_ = 0.0047, *H*_d_ = 0.6681). Analysis of the differences in genetic diversity between the extent geographic distributions is the major method for reconstructing species histories during the Quaternary (Jakob, Martinezmeyer, & Blattner, 2009). As found in northern Europe, the recently colonized territories from multiple glacial refugia show rather increased genetic diversity due to lineage admixture. Groups with high levels of genetic diversity provide strong evidence for the existence of multiple glacial refugia for cool‐temperate forest trees (Widmer & Lexer, 2001), this result did not support the possibility that the northern populations migrated from the south during the LGM.

Furthermore, a significantly higher *N*_ST_ over *G*_ST_ was found between DB and NF based on the cpDNA data, which indicated the presence of phylogeographic structure and that the populations are strongly differentiated genetically and there was no extensive migration between the DB and NF groups after divergence (Pons & Petit, 1996).

Geographically, the DB group mainly occurred north of 40°N. The ENM estimates based on the CCSM and MIROC models showed that, at the LGM, LIG, and probably other glacial periods, *P. davidiana* mainly persisted in the Hengduan Mountains and areas around the Qinling Mountains (Figure 4). In northeast of China, it was estimated that there was also a suitable distribution area for *P. davidiana*, but its distribution range has slightly expanded under the present climate (Figure 4). The genetic bottlenecks due to range expansions may have been buffered by long‐distance pollen and seed dispersal via wind in *Populus* species. This agreed with the results of the Bayesian skyline plots and neutrality tests.

Therefore, the present populations of northeast China most likely came from local refugia, rather than the colonist from the south populations. These results all showed that at least one refugium of *P. davidiana* might have existed in northeast China in the LGM.

### 4.2 Glacial refugia in southwestern China

From the results of haplotype variation, distribution, and the network of cpDNA, the older distinctive haplotype (H32) only existed in the NF region and the ancient haplotype H5 mainly appeared in southwest China (Figure 1). The diversity of haplotypes and nucleotides for the cpDNA sequence and nuclear locus in the NF region was also high (Table 2). The ENM estimates based on the CCSM and MIROC models suggested that during the LGM and LIG periods, the Hengduan Mountains had a large suitable area for *P. davidiana* (Figure 4). Based on the phylogeographical studies on a number of endemic species in southern China, Hwang *et al.* (Hwang et al., 2003) inferred several refugia in southern China, while Shen *et al.* also found possible refugia of *Ginkgo* *biloba* in southwestern China (Hwang et al., 2003; Shen et al., 2005). In addition, the Hengduan range and the Lingnan region were normally considered to be the most likely refugia for plant species in southern China, as this region had high levels of plant diversity and endemism in China and many ancient plant species were also found in the region (Shen et al., 2005; Ying, 2001). Moreover, the NF region is rich in topography, climate, and ecological conditions and was spared from the direct effects of the repeated Pleistocene continental glaciation, providing many appropriate environment conditions for the species in the LGM (Liu & Basinger, 2000). Thus, the Hengduan range was most likely to be a refugium for *P. davidiana* according to the results of our research.

### 4.3 Possible reasons for the higher genetic diversity level of *P. davidiana* populations in central China

According to the results, the frequencies and compositions of the HB regions were much different from those in the DB and NF regions. They had their own private cpDNA haplotypes, and all the region had higher average levels of haplotype and nucleotide diversity (Figure 1 and Table 2). As pointed out by Wright & Gaut (Wright & Gaut, 2005) and Wen *et al.* (Wen, Han, & Shun, 2010), several factors may contribute to nucleotide diversity in plant species and populations, such as natural selection, demographic population history, and mode of reproduction. Natural selection can be ruled out based on the neutrality test of nuclear loci apart from DSH21. Therefore, we speculated that there might be one or more refugia in these areas and these regions have abundant species and a large number of endemic genera of plants (Ying, 2001), maintaining high effective population sizes that result in high levels of nucleotide diversity (Ismail et al., 2012). This hypothesis was also supported by the results of the ENM estimates based on the CCSM and MIROC models, indicating that suitable distribution areas may have existed in the Qinling and Helan Mountains and their surrounding ranges during the LGM. On the other hand, the HB region also had higher haplotype and nucleotide diversity, which might be explained as a result of the crossing of populations around the HB region. As speculated in the study by Zeng, Liao, Petit, and Zhang (2010), this region might be an intersection of gene flow between northern and southern populations of *P. davidiana*.

### 4.4 The main reason for the high differentiation of the three defined groups: DB, HB, and NF

Three main phylogeographical groups (DB, HB, and NF) were uncovered in the distribution of *P. davidiana*, according to the results of population genetic structure and the variation and distribution of nuclear haplotypes based on the fragment from the DSH3, 5, 6, 7, 12, 21 loci. The pairwise *F*_ST_ values showed strong genetic differentiation among groups (0.192–0.362). These results together with the AMOVA analysis indicated high genetic differentiation between pairwise groups

The pattern of genetic differentiation during ecological speciation is shaped by a combination of evolutionary forces. Processes such as genetic drift, local reduction of gene flow around genes causing reproductive isolation, hitchhiking around selected variants, variation in recombination and mutation rates are factors that can contribute to the heterogeneity of genetic differentiation (Feulner et al., 2015). For the widely distributed species, genetic variation among populations mainly caused by geographic isolation and different climate conditions. Gene flow can increase genetic variation within populations and reduce divergence among populations, leading to populations tend to be consistent. However, *P. davidiana *is a wind‐pollinated species and there were several mixed zones of populations in DB, HB, and NF groups according to our study. It was surprise that genetic variation still significantly existed between these regions, indicating that geographic isolation might not be the main reason to produce genetic differentiation among the three groups. On the other hand, the populations of *P. davidiana* in SOUTH (excluding GBJD population) group mainly distributed in south of Sichuan Province, west of Guizhou Province and Yunnan Province (Table S1), which belong to the southern subtropical areas. The climatic characteristics in these areas such as altitude, temperature, humidity, illumination, and accumulated temperature are different from those in northern subtropical. It was probably because of the differences of climate among these areas, and different adaptabilities lead to the high differentiation of the three groups.

From the phylogeographic study of *P. davidiana*, we clearly demonstrated that refugia most likely existed in northeastern and southwestern China, and this is important for revealing the relationships of different fauna and flora, understanding the mechanism of species formation, and protecting the diversity of species in China. These findings have implications for rethinking the refugia of other temperate forest species in East Asia as well as the role of these local northern refugia in maintaining high species diversity in this region.

## CONFLICT OF INTEREST

The authors declare that the research was conducted in the absence of any commercial or financial relationships that could be construed as a potential conflict of interest.

## AUTHOR CONTRIBUTIONS

Z.H performed the experiments and wrote the study, Z.S.W. and J.G.Z. designed the research. Z.Y.Y and S.H.D made valuable suggestions on a preliminary version of this study.

## DATA ACCESSIBILITY

The new sequences were deposited in GenBank under accession numbers (MG287147–MG288502, MG345155–MG346171, MG426214–MG427048).
