# Supplementary material for: Phylogeographic analyses of a widely distributed Populus davidiana: Further evidence for the existence of glacial refugia of cool‐temperate deciduous trees in northern East Asia
Source: Ecol Evol. 2018 Dec 11;8(24):13014–26. doi: 10.1002/ece3.4755 (PMC6308874; doi:10.1002/ece3.4755)
Supplement: Supplementary file 1 [file ECE3-8-13014-s001.docx]

**Appendix**

Additional information may be found in the online version of this article.

**Table S1** Information on sampling locations

**Table S2** Primers used in this research

**Table S3** 19 bioclimatic parameters were selected to generate the distribution model

**Table S1.** Information on sampling locations

| Population number | Location | Voucher information | Sample size | Altitude(m) | Longitude | Latitude | Haplotype  (Individuals) |
| --- | --- | --- | --- | --- | --- | --- | --- |
| TXSY | Mohe | Zhang Jianguo 1 | 30 | 546 | E 122°49′ | N 52°54′ | H1(18), H2(2), H3(5), H4(1), H5(4); |
| HH | Heihe | Wang Zhaoshan 1 | 17 | 124 | E 127°30′ | N 50°14′ | H1(13), H3(3), H6(1); |
| HS | Heishui | Zhang Jianguo 2 | 5 | 129 | E 124°59′ | N 45°42′ | H7(3), H8(1), H9(1); |
| SYJY | Jiayin | Zhang Jianguo 3 | 7 | 139 | E 130°24′ | N 48°52′ | H11(7); |
| SYTW | Tangwang | Zhang Jianguo 4 | 6 | 395 | E 129°34′ | N 48°27′ | H1(2), H12(1), H13(1), H14(2); |
| HN | Huanan | Zhang Jianguo 5 | 19 | 170 | E 130°33′ | N 46°14′ | H1(10), H3(9); |
| DL  DLSY | Haerbin  Yichun | Zhang Jianguo 6  Zhang Jianguo 7 | 5  6 | 251  221 | E 129°01′  E 129°02′ | N 47°01′  N 47°03′ | H1(1), H3(9);  H1(2), H2(3), H10(1); |
| MRS | Maoershan | Wang Zhaoshan 2 | 11 | 253 | E 127°31′ | N 45°17′ | H1(1), H3(5), H41(1), H42(1), H43(1), H44(1), H45(1); |
| TH | Tahe | Wang Zhaoshan 3 | 20 | 358 | E 109°12′ | N 50°0′ | H1(16), H3(3), H46(1); |
| LHS | Lianhuashan | Wang Zhaoshan 4 | 20 | 225 | E 125°74′ | N 43°86′ | H1(8), H2(2), H3(5), H5(2); |
| FS | Fushun | Wang Zhaoshan 5 | 20 | 83 | E 123°96′ | N 41°88′ | H1(6), H2(2), H3(4), H5(2); |
| WC | Chengde | Zhang Jianguo 8 | 27 | 904 | E 117°44′ | N 41°56′ | H1(6), H3(1), H5(16); |
| HL | Hualin | Zhang Jianguo 9 | 8 | 251 | E 116°03′ | N 39°57′ | H5(4), H18(1), H19(3); |
| XLM | Xiaolongmen | Wang Zhaoshan 6 | 22 | 809 | E 115°26′ | N 39°57′ | H1(1), H3(3), H5(12), H18(4), H19(2), H20(2); |
| BJ | Beijing | Wang Zhaoshan 7 | 5 | 139 | E 116°38′ | N 40°10′ | H5(5); |
| GS  XLS | Gansu  Xinglongshan, | Zhang Jianguo 10  Zhang Jianguo 11 | 20  23 | 1171  1525 | E 105°43′  E 104°04′ | N 34°34′  N 38°39′ | H5(16), H21(1), H22(2), H23(1);  H5(6), H48(16), H49(1); |
| HLS | Helanshan | Zhang Jianguo 12 | 10 | 1310 | E 116°04′ | N 38°50′ | H24(1), H25(2), H26(1), H27(1), H28(5); |
| WT | Wutai | Zhang Jianguo 13 | 26 | 1032 | E 113°26′ | N 38°73′ | H5(23), H19(2), H47(1); |
| XN | Xiangning | Wang Zhaoshan 8 | 5 | 961 | E 110°85′ | N 35°97′ | H5(2), H19(5), H47(1), H48(1); |
| HEN | Henan | Zhang Jianguo 14 | 18 | 486 | E 111°03′ | N 34°32′ | H5(3), H18(1), H19(13), H29(1); |
| BTM | Baotianman | Zhang Jianguo 15 | 17 | 350 | E 112°11′ | N 33°57′ | H5(2), H18(5), H19(5), H40(3); |
| WS | Wushan | Zhang Jianguo 16 | 11 | 409 | E 109°49′ | N 31°10′ | H5(1), H18(5), H19(2), H30(1), H31(2); |
| ZGN | Zagenao | Wang Zhaoshan 9 | 24 | 1881 | E 103°10′ | N 31°26′ | H5(15), H36(9); |
| DXP | Daxiangping | Wang Zhaoshan 10 | 12 | 2268 | E 102°19′ | N 27°31′ | H5(1), H19(1), H32(10); |
| JBG | Jiaobangou | Zhang Jianguo 17 | 19 | 1910 | E 102°06′ | N 27°25′ | H5(3), H19(9), H32(5), H33(2); |
| SYBJ | Bijie | Zhang Jianguo 18 | 12 | 1517 | E 105°17′ | N 27°17′ | H5(3), H19(4), H32(5); |
| WN | Weining | Zhang Jianguo 19 | 24 | 2249 | E 104°16′ | N 26°51′ | H5(6), H19(11), H32(7); |
| KM1 | Kunming | Zhang Jianguo 20 | 12 | 1927 | E 102°53′ | N 24°52′ | H5(11), H19(10); |
| DAL | Dali | Zhang Jianguo 21 | 10 | 2353 | E 100°10′ | N 25°37′ | H5(6), H19(1), H32(3), H34(1), H35(3); |
| JC | Jianchuan | Zhang Jianguo 22 | 14 | 2207 | E 99°54′ | N 26°32′ | H19(7), H32(3); |
| GBJD | Gongbujiangda | Zhang Jianguo 23 | 8 | 4155 | E 93°14′ | N 29°53′ | H36(1), H37(6), H38(1) |

| **Table S2**. Primers used in this research | | |
| --- | --- | --- |
| locus | Primer sequences (5'-3') | Ta (℃) |
| nuclear |  |  |
| DSH3 | F: TCTGCTTTCCACTTCTTGC | 55 |
|  | R: CATACTCTCCCATTGTCCC |  |
| DSH5 | F: TGGCAGAATCACCAGACCCTC | 59 |
|  | R: CCAATTTAGCATCTTCAGCCTCAT |  |
| DSH6 | F: GCCTCCTGATTATTATGC | 54 |
|  | R: TATTACAAGCCCTTCCAG |  |
| DSH7 | F: GTTTGTTGTTCTGTTGATTGT | 56 |
|  | R: GGCTTCTCTTCTCTGATATTT |  |
| DSH12 | F: CACCACATCCCGCTTTCTCTCTTCACTT | 57 |
|  | R: TAAACCCCAGGAGGCAAAACAGCACCAG |  |
| DSH21 | F: CATGCTTATGAAGGTGTGGGCTT | 53 |
|  | R: TGCAAACATCTCACTGGTGACTG |  |
| cpDNA |  |  |
| *trnK* | F:GGGTTGCCCGGGACTCGAAC | 55 |
|  | R:ATTGGATTTGCTGTGATA |  |
| *trnL-trnF* | F:CGAAATTGGTAGACGCTACG | 54 |
|  | R:ATTTGAACTGGTGACACGAG |  |
| *atpI* | F:CCAACCCAGCAGCAATAAC | 56 |
|  | R:TATTTACAAGTGGTATTCAAGCT |  |

**Table S3** 19 bioclimatic parameters were selected to generate the distribution model

| climatic variables | Discription |
| --- | --- |
| 1 | Annual mean temperature |
| 2 | Mean diurnal range |
| 3 | Lsothermality |
| 4 | Temperature seasonality |
| 5 | Max temperature of warmest month |
| 6 | Min temperature of coldest month |
| 7 | Temperature annual range |
| 8 | Mean temperature of wettest quarter |
| 9 | Mean temperature of driest quarter |
| 10 | Mean temperature of warmest quarter |
| 11 | Mean temperature of coldest quarter |
| 12 | Annual precipitation |
| 13 | Precipitation of wettest month |
| 14 | Precipitation of driest month |
| 15 | Precipitation seasonality |
| 16 | Precipitation of wettest quarter |
| 17 | Precipitation of driest quarter |
| 18 | Precipitation of warmest month |
| 19 | Precipitation of coldest month |
